# The Buffering Effect of Workplace Resources on the Relationship between the Areas of Worklife and Burnout

**DOI:** 10.3389/fpsyg.2017.00012

**Published:** 2017-01-17

**Authors:** Paul Jimenez, Anita Dunkl

**Affiliations:** Department of Psychology, University of GrazGraz, Austria

**Keywords:** burnout, longitudinal, strain, stress, workplace resources

## Abstract

**Background:** Workplace resources are found to play a major role in the stress–strain relationship. However, usually different types of resources are investigated, whereas investigating different facets of stress (“stressors”) receive less attention in research about the relationship between stress, strain and resources. Based upon recent research, we expected that workplace resources moderate the relationship between stressors (operationalized with the areas of worklife) and long-term strain (operationalized with three dimensions of burnout) in the sense that workplace resources buffer the negative effects of stressors on strain.

**Method:** Hypotheses were tested in a longitudinal sample of 141 Austrian workers, who participated two times in an online study over a period of 6 months. Hierarchical multiple regression analysis was used to test the proposed relationships.

**Results:** The results imply that workload and reward seem to be the most important predictors for burnout. Workload is important for emotional exhaustion, whereas reward is important for cynicism. Value-fit at the workplace plays a significant role for cynicism, but only if resources at the workplace are high. Further moderating effects of resources were found for the outcome personal accomplishment. More specifically, results indicate that having high resources in a high workload environment increases personal accomplishment after a time interval of 6 months. In addition, employees experiencing high levels of control but low workplace resources show less personal accomplishment.

**Conclusion:** Despite the limiting aspects of the relatively short period of time we can see that resources can buffer workload effects. This should be taken into consideration when doing risk assessments in practice as work design should focus on resources even more when high workload can be found.

## Introduction

Workplace resources have an essential influence on the stress–strain relationship. Previous studies on buffering effect of resources on the stress–strain relationship mainly used a summarized factor of stress (e.g., high job demands) to predict negative work-related outcomes such as strain. This more global view of stress only scratches the surface in research on the stress–strain relationship. However, authors emphasize that different types of stress have to be analyzed to get a better understanding about stress and strain (Bakker et al., 2005). Paradoxically, research around the stress–strain framework tends to investigate the moderating effects of different types of resources (e.g., social support, feedback, latitude) more deeply than the effects of different stress factors (e.g., Xanthopoulou et al., 2007; van den Tooren and de Jonge, 2012). Our study focuses on this complex subject and investigates the effects of different facets of stress (as we will call it later on synonymously stressors) and their interaction with workplace resources on strain.

### Stress and Strain at the Workplace

In the long tradition of stress research, the terms stress and strain are often used interchangeably. A strict way to differentiate between them was introduced in the norm series of the ISO 10075 (ISO, 2000a,b, 2015). As we can see norms as the current status of scientific knowledge we therefore refer to these definitions in this text. The norm ISO 10075-1 (ISO, 2000a, p. 3; s.a. Demerouti et al., 2002; Zoni and Lucchini, 2012), defines mental stress and strain as follows: mental stress is the “*…total of all assessable influences impinging upon a human being from external sources and affecting that person mentally*” and mental strain is defined as the “*…immediate effect of mental stress within the individual depending on their current condition.*” Thus, the norm summarizes the objective stressors under the term “stress” and the outcome of these stressors (e.g., the individual evaluation of these stressors) as “strain.” The consequences of strain can be further differentiated into short-term and long-term effects of strain. Short-term effects compass mental fatigue, monotony, satiation and stress sensations (Demerouti et al., 2002). Long-term effects of strain result from repeated exposure to strain. One important chronic reaction to prolonged impairing strain would be burnout which is now mentioned especially in the upcoming norm of the ISO 10075-1 (ISO, 2015).

The definition of stress is similar to the definition of “job demands” (Bakker et al., 2005), as job demands are described as physical, mental, social, or organizational aspects of the job that require sustained effort/skills and are associated with physiological and psychological costs (as in the concept of Meijman and Mulder, 1998). Bakker et al. (2005) emphasize that job demands are not necessarily negative and can influence positive outcomes (e.g., engagement) as well.

Stress is a neutral term and does not bear a negative (or positive) connotation. In the ISO 10075-1 (ISO, 2000a), stress is seen as a total value that results by summarizing all influences at the workplace – whether they have negative effects or not. However, it is possible to distinguish different stressors that might have different short-term or long-term outcomes. Rohmert (1984) distinguishes quantifiable and non-quantifiable stressors. In his view, quantifiable stressors compasses noise, temperature or air pressure. Non-quantifiable stressors usually results from working tasks and includes time pressure, monotony or responsibility.

One classification of stressors is stated in the ISO 10075-1 (ISO, 2000a, p. 4), which categorizes stress in following categories: task requirements, physical conditions, social and organizational factors, and societal factors. The ISO concept therefore focuses to a system of stressors which can lead to a variety of possible outcomes, negative but also positive ones. The main strength of this model lies in the strict distinction between stressors and the effects. As positive outcomes (e.g., practice effects or competence development) are also possible it is not meaningful to classify stressors as “good” or “bad,” the consequences of stress are “depending on an individual’s personal resources and his/her perception of the situation” (Demerouti et al., 2002, p. 425).

A framework that compasses different stressors which are seen as potential risk factors especially for burnout can be seen in the concept of the areas of worklife (Maslach and Leiter, 2008). In this concept, six domains of the work environment have been identified that can serve as “organizational risk factors” (Maslach and Leiter, 2008, p. 500) for negative work-related outcomes, such as burnout: workload, control, reward, community, fairness, and values.

The area of workload is described as experiencing qualitative and quantitative work overload that depletes the person’s capacity to meet the demands of the job. Control means having sufficient latitude at work and having possibilities to make important decisions. Reward refers to financial and non-financial reward (e.g., recognition from colleagues, supervisors and clients) for the employees’ work contributions. Community is the overall quality of social interactions of work. The area of fairness describes the extent to which decisions at work are perceived as fair. Finally, values (or value-congruence) describes the match of the employees’ and organizations’ job goals and expectations (a full description can be found, e.g., in Maslach and Leiter, 2008).

In all six areas, discrepancies between person and work environment can occur which are described as mismatch (Maslach and Leiter, 2008). In other words, these six areas are able to reveal critical work conditions. On the other hand, they serve as prevention factors for negative work-related outcomes if they are evaluated positively (Laschinger et al., 2015; Boamah and Laschinger, 2016). Especially the area of values is found to have an essential effect on work-related outcomes such as burnout (emotional exhaustion, cynicism, and personal accomplishment) and turnover intention (Leiter and Maslach, 2005, 2009; Leiter et al., 2009). A conflict between individual and organizational values leads to less engagement with the tasks, as work is perceived as personally irrelevant (Leiter and Maslach, 1999). This reduced involvement depletes the employees’ energy and contributes to exhaustion and cynicism.

The areas of workload and reward are important predictors for emotional exhaustion and cynicism, respectively (Leiter and Maslach, 2009). A lack of (financial or non-financial) reward leads to feelings of inefficacy and meaninglessness and this in turn contributes to cynicism (Cartwright and Holmes, 2006). High workload prevents employees from adequate recovery which is a critical factor for fatigue and exhaustion (Sonnentag et al., 2010). Especially the relationship between workload and emotional exhaustion is rather persistent and stable over time, which means that high workload at one point is able to predict emotional exhaustion even after a long period of time (Michielsen et al., 2004).

The extent of short-term and long-term outcomes of stress depends on moderating variables, such as personal or workplace resources. In the ISO 10075-1 (ISO, 2000a), the current condition of the individual is highlighted, which is dependent on the individual’s workplace resources. Next to individual resources, workplace resources (or “job resources”) have received much attention in research about outcomes of stress (Karasek, 1979; Bakker et al., 2005). Indeed, targeting workplace resources is a sustainable way to prevent negative outcomes of stress as workplace resources seem to be the precursor of individual resources such as employees’ self-efficacy, self-esteem, and optimism (Xanthopoulou et al., 2007).

### The Role of Resources at the Workplace

Resources in the working context refer to the physical, psychological, social, or organizational aspects of the job that are able to reduce job demands, stimulate personal growth, learning and development and/or are functional in achieving work goals (Bakker and Demerouti, 2007). Resources at the workplace play a major role in the relationship between stress and strain (Demerouti et al., 2002) and have been studied in a variety of different frameworks: the demand-control model (Karasek, 1979), the effort-reward imbalance model (Siegrist, 1996), the job demands-resources (JD-R) model (Bakker and Demerouti, 2007), and the resources/recovery-stress model (Kallus and Kellmann, 2000; Kallus, 2016). Following the demand-control model, aspects of job control (e.g., latitude, autonomy) buffer the effect of stress on strain. In the effort-reward imbalance model, the important job resource is reward (e.g., salary, promotion, job security or esteem reward) that may buffer the critical relationship between stress and strain.

The important role of resources is also illustrated in Hobfoll’s (1989) conservation of resources (COR) model. This model proposes that strain is a result of a threat to resources, actual loss of resources or insufficient gain of additional resources. Any of these three paths can cause strain and might lead to burnout over time.

In the JD-R model (Bakker and Demerouti, 2007), negative outcomes of stress can be decreased if job resources are high. In contrast to Karasek’s (1979) or Siegrist’s (1996) model, the JD-R model does not limit resources to one aspect (e.g., job control or reward) but expands the view of resources to a variety of states. Indeed, examples of job resources can be found on many levels, such as on the organizational level (e.g., career opportunities, salary), on the interpersonal level (e.g., social support from colleagues or supervisor) or on the task/work level (e.g., skill variety, participation possibilities) (Bakker et al., 2007). When a sufficient amount of job resources is available, it is even possible to have positive effects of stress in the sense of challenging jobs (Bakker et al., 2010). This again is in line with the model in the ISO 10075-1.

Kallus’ (2016) resources/recovery-stress model assumes that workplace stress can be especially harmful and lead to negative outcomes if the relation between stress and recovery/resources is imbalanced. In his view the terms recovery and resources are used as nearly interchangeably. If a person experiences high stress, this stress can lead to strain depending on the person’s individual resources and depending on the person’s recovery processes to strengthen resources. In this model, resources are able to buffer the relation between stress and strain only if they have been recovered. This view is also shared by Oerlemans and Bakker (2014), where resources have to be replenished regularly to show stress-reducing effects. Therefore, the availability and state of resources must increase to the same extent as stress to cope successfully with the situation. This is in line with the COR model, where negative outcomes occur if work demands are high and resources have not been adequately replenished (Freedy and Hobfoll, 1994). Especially resources on the task level (latitude and autonomy) and on the interpersonal level (social support) have been found to reduce the negative effects of stress on strain (Halbesleben, 2006; Nahrgang et al., 2011). In the present study, we conceptualize workplace resources as a combination of task level and interpersonal level resources.

### Theoretical Model and Hypotheses

The research reported in this article used a longitudinal research design to investigate the relationship between stress, workplace resources and outcomes of stress. To measure stress, we use the concept of the areas of worklife (Maslach and Leiter, 2008), which is a framework that compasses six different stressors (workload, control, reward, community, fairness, and values). Stress is often measured as a total value by summarizing many workplace stressors (e.g., as “demands,” Hakanen et al., 2008; Trépanier et al., 2014). In our study we want to measure stress deeper in different facets; therefore, we focus on stressors described in the concept of the areas of worklife. In addition, we analyze the effects of stress at two time points (after 6 months), thus testing the causal relationship between stress and strain.

According to the ISO 10075-1 (ISO, 2000a), the immediate outcome of stress would be strain. However, we are more interested in the long-term effects of stress; therefore, the concept of burnout is used to operationalize the outcome of stress, which is a result from repeated exposure to strain (ISO, 2000a; Demerouti et al., 2002).

On basis of the studies conducted by Leiter and Maslach (2005, 2009) regarding the areas of worklife, following hypotheses are stated:

H1: The area of workload is a significant predictor for Emotional Exhaustion.
H2: The area of reward is a significant predictor for Cynicism.
H3: The area of values is negatively associated with Emotional Exhaustion and Cynicism and positively associated with Personal Accomplishment.

In line with the JD-R model and the resources/recovery-stress model, we hypothesize that workplace resources are negatively related to burnout and are able to buffer the negative effect of stressors (measured with the six areas of worklife) on burnout.

H4: Workplace resources are negatively related to emotional exhaustion and cynicism and positively related to personal accomplishment.
H5: Workplace resources moderate the negative relation between stressors (measured with the areas of worklife) and emotional exhaustion/cynicism such as the relationship is weaker for employees with high workplace resources.
H6: Workplace resources moderate the positive relation between stressors (measured with the areas of worklife) and personal accomplishment such as the relationship is stronger for employees with high workplace resources.

The main strengths of the present study lie in the differentiated view of stress by investigating the effects of different stressors and in the longitudinal design to detect causal relationships. In addition, moderating effects of workplace resources are tested cross-sectionally and longitudinally to gain better insight in the short-term and long-term buffering role of workplace resources in the stress–strain relationship.

## Materials and Methods

### Participants and Procedure

The data were collected as part of a larger longitudinal study conducted among Austrian workers. For this study, persons were recruited from other studies executed at the Department of Psychology at the University of Graz and were asked for their consent to contact them again for future studies. The approval of the ethical commission was obtained before the start of the whole longitudinal study. The first measurement took place in spring and the second in autumn. The measurement times were carefully chosen to avoid holiday seasons. The time interval between the single measurement points was 6 months. The two measurement points are referred to as Time 1 (T1) and Time 2 (T2).

At T1, 626 participants filled-in all questionnaires in the online survey. At T2, 439 participants took part. The final sample consisted of 141 participants that took part at both measurement points and also were at the same workplace at both times. Of these 141 respondents, 61% were female (male: 39%) and their mean age was 43.7 years (*SD* = 9.04). The majority worked full-time or more (83.7%), 16.3% worked part-time. The participants worked in different industrial sectors, mostly from health sector (19.9%), manufacturing (12.1%), public sector (11.3%), and general services (9.2%). To analyze a possible drop-out bias, participants who participated in T1 were compared to those who participated in both waves. The analysis revealed that both groups did differ significantly in their experience of workplace resources (*p* < 0.05). More specifically, workplace resources were higher in the group that participated in both waves compared to participants who participated only in T1. For all other variables, no significant effects were found.

### Measures

#### Areas of Worklife

The Areas of Worklife Scale (AWS; Leiter and Maslach, 1999) measures six different areas of worklife: (1) workload, (2) control, (3) reward, (4) community, (5) fairness and (6) values. The participants are asked to answer 29 items on a 5-point Likert-Scale ranging from 1 (strongly disagree) to 5 (strongly agree). One example item for the area of control is “I have professional autonomy/independence in my work” and one example item for the area of values is “My values and the organization’s values are alike.” The German translation by Schulze (see Brom et al., 2015) was used in this study.

#### Workplace Resources

The Recovery-Stress-Questionnaire for Work (RESTQ-Work; Jiménez and Kallus, 2016) assesses different aspects of stress and recovery activities and states in the past 7 days/nights. In the present study, the short version of the RESTQ-Work (RESTQ-Work-27) with 27 items was used. The items can be assigned to a stress or recovery/resources score. In the present study, only the workplace resources score without recovery aspects was analyzed (Jiménez et al., 2016a). The workplace resources score consists of three sub-dimensions, each measuring another type of work-related resources (leisure/breaks, psychosocial resources, work-related resources). The RESTQ-Work-27 allows giving feedback by using the stress and resources scores. The underlying sub-dimensions can only be used for screening purposes in the practical field (Jiménez et al., 2016a). One example item for the resources score is “In the past 7 days/nights… I was able to relax during my breaks” or “In the past 7 days/nights… I had the chance to work on a variety of tasks.” The answer scale is a 7-point-Likert-Scale ranging from 0 (never) to 6 (always).

#### Burnout

The Maslach-Burnout-Inventory – General Survey (MBI-GS; Schaufeli et al., 1996) measures burnout with three dimensions: emotional exhaustion, cynicism, and personal accomplishment. In the present study, the German version of the MBI-GS by Büssing and Glaser (1998) was used. The 16 items can be answered on a 7-point-Likert-Scale ranging from 0 (never) to 6 (every day).

### Analysis

With regard to analyses, we used bivariate correlation and hierarchical regression analyses. Hierarchical multiple linear regression analyses were performed for each dependent variable (emotional exhaustion, cynicism, personal accomplishment) and were conducted for the cross-sectional sample (at T1) and the longitudinal sample. In the longitudinal analysis, the dependent variable at T1 was controlled for all outcomes. To analyze the interaction between the variables, the interaction terms first were mean-centered (z-transformed) to minimize multicollinearity and then entered in the regression analysis. The data was analyzed using SPSS Version 22.

## Results

### Descriptive Statistics

Descriptive statistics (means and standard deviations) all study variables are shown in **Table 1**. The descriptive statistics were calculated for both measurement points separately. The data for workplace resources and the three burnout dimensions were compared to the data of a representative Austrian sample, collected in 2015 (Jiménez et al., 2016b). Compared to the representative Austrian sample, the study sample showed higher values in workload, emotional exhaustion and cynicism and lower values in fairness and workplace resources (see **Table 1**). Intercorrelations between variables for T1 and T2 (cross-sectional and longitudinal) as well as internal consistencies (Cronbach’s Alpha, α) are shown in **Table 2**.

**Table 1:** Means (M) and standard deviations (SD) of all study variables.

Variable	Time 1 (T1)				Time 2 (T2)				Austrian representative sample	
			
	Longitudinal sample (*N* = 141)		All responses (*N* = 468)		Longitudinal sample (*N* = 141)		All responses (*N* = 402)		(*N* = 1200)	
					
	*M*	*SD*	*M*	*SD*	*M*	*SD*	*M*	*SD*	*M*	*SD*
Workload	3.25	0.92	3.35	0.90	3.23	0.96	3.34	0.99	2.76	0.88
Control	3.46	0.89	3.45	0.98	3.30	1.10	3.38	1.03	3.40	1.03
Reward	3.07	0.86	3.07	0.84	2.88	0.83	2.95	0.87	3.32	0.98
Community	3.28	0.87	3.23	0.82	3.52	0.80	3.33	0.88	3.34	0.80
Fairness	2.85	0.93	2.89	0.85	2.77	0.94	2.77	0.90	3.05	0.80
Values	3.45	0.81	3.38	0.84	3.31	0.71	3.31	0.79	3.44	0.81
Workplace resources	2.96	0.98	2.87	0.97	3.08	1.11	3.08	1.09	3.28	1.16
Emotional exhaustion	3.83	1.22	3.95	1.20	3.76	1.24	3.87	1.22	3.23	1.30
Cynicism	3.34	1.26	3.38	1.25	3.36	1.34	3.28	1.33	2.97	1.28
Personal accomplishment	4.66	0.86	4.61	0.84	4.62	0.83	4.61	0.82	4.71	0.83


*Note 1: areas of worklife at T2: *N* = 148 (all responses) and *N* = 53 (longitudinal sample).*

**Table 2:** Correlations and internal consistencies (Cronbach’s alpha) between all study variables (Time 1 and Time 2; *N* = 141).

Variable	1	2	3	4	5	6	7	8	9	10	11	12	13	14	15	16	17	18	19	20
Time 1 (T1)																				
(1) Workload	0.85																			
(2) Control	-0.33***	0.80																		
(3) Reward	-0.39***	0.36***	0.83																	
(4) Community	-0.32***	0.27***	0.41***	0.88																
(5) Fairness	-0.29***	0.43***	0.45***	0.50***	0.89															
(6) Values	-0.28***	0.39***	0.49***	0.45***	0.74***	0.81														
(7) Workplace resources	-0.44***	0.55***	0.63***	0.53***	0.51***	0.45***	0.87													
(8) Emotional exhaustion	0.65***	-0.45***	-0.57***	-0.27***	-0.38***	-0.40***	-0.61***	0.91												
(9) Cynicism	0.53***	-0.47***	-0.70***	-0.46***	-0.56***	-0.59***	-0.67***	0.71***	0.86											
(10) Personal accomplishment	-0.25***	0.48***	0.62***	0.33***	0.44***	0.50***	0.65***	-0.52***	-0.67***	0.86										
Time 2 (T2)																				
(11) Workload	0.65***	-0.13	-0.02	-0.03	-0.06	0.00	-0.02	0.31*	0.21	0.06	0.86									
(12) Control	-0.17	0.60***	0.13	0.23	0.29*	0.29*	0.50***	-0.30*	-0.36**	0.39**	-0.37**	0.89								
(13) Reward	-0.10	0.23	0.53***	0.04	0.33*	0.37**	0.30*	-0.17	-0.36**	0.33*	-0.14	0.34*	0.73							
(14) Community	-0.13	0.26	0.10	0.67***	0.37**	0.41**	0.45***	0.05	-0.14	0.32*	-0.20	0.41**	0.22	0.89						
(15) Fairness	-0.15	0.42**	0.40**	0.33*	0.81***	0.62***	0.47***	-0.27	-0.56***	0.54***	-0.23	0.53***	0.45***	0.42**	0.88					
(16) Values	-0.09	0.34*	0.14	0.20	0.56***	0.66***	0.29*	-0.03	-0.46***	0.30*	-0.30*	0.55***	0.44**	0.42**	0.68***	0.72				
(17) Workplace resources	-0.36***	0.41***	0.53***	0.44***	0.46***	0.43***	0.75***	-0.57***	-0.52***	0.56***	-0.17	0.76***	0.39**	0.55***	0.52***	0.49***	0.91			
(18) Emotional exhaustion	0.58***	-0.37***	-0.47***	-0.24***	-0.38***	-0.38***	-0.47***	0.73***	0.55***	-0.40***	0.66***	-0.48***	-0.38*	-0.22	-0.29*	-0.38**	-0.60***	0.91		
(19) Cynicism	0.39***	-0.36***	-0.56***	-0.30***	-0.49***	-0.53***	-0.52***	0.60***	0.66***	-0.48***	0.41**	-0.67***	-0.59***	-0.37**	-0.59***	-0.66***	-0.68***	0.73***	0.92	
(20) Personal accomplishment	-0.17*	0.30***	0.45***	0.20*	0.30***	0.35***	0.45***	-0.35***	-0.40***	0.67***	-0.12	0.65***	0.49***	0.48***	0.52***	0.52***	0.68***	-0.47***	-0.62***	0.84


*^∗^*p* < 0.05, ^∗∗^*p* < 0.01, ^∗∗∗^*p* < 0.001; Cronbach’s alpha (α) in the diagonal; areas of worklife at T2: *N* = 53.*

### Cross-Sectional Sample

To test the proposed hypotheses, hierarchical multiple linear regression analyses were conducted for the cross-sectional sample (at T1) and the longitudinal sample. In **Table 3**, the results for T1 are depicted. In the first step, the areas of worklife were stepped into the equation, which was significant for all models (Δ*R*^2^_emotional exhaustion_ = 0.55; Δ*R*^2^_cynicism_ = 0.57, Δ*R*^2^_personal accomplishment_ = 0.37). The second step included workplace resources, which significantly accounted for an additional variance for all outcomes (Δ*R*^2^_emotional exhaustion_ = 0.04; Δ*R*^2^_cynicism_ = 0.05, Δ*R*^2^_personal accomplishment_ = 0.07). The third step included the interaction terms, which were non-significant for all models.

**Table 3:** Cross-sectional, hierarchical multiple regression analysis for all predicted variables at Time 1 (T1).

	Emotional exhaustion T1			Cynicism T1			Personal accomplishment T1		
			
Step and variable	β	*p*	Δ*R*^2^	β	*p*	Δ*R*^2^	β	*p*	Δ*R*^2^
Step 1: Areas of worklife			0.55***			0.57***			0.37***
Workload T1	0.50***	<0.001		0.16***	<0.001		-0.02	0.663	
Control T1	-0.01	0.829		-0.02	0.555		0.06	0.184	
Reward T1	-0.16***	<0.001		-0.27***	<0.001		0.23***	<0.001	
Community T1	0.00	0.985		-0.01	0.853		-0.03	0.465	
Fairness T1	0.07	0.131		-0.01	0.898		-0.10*	0.050	
Values T1	-0.10*	0.017		-0.25***	<0.001		0.22***	<0.001	
Step 2:			0.04***			0.05***			0.07***
Workplace resources T1	-0.26***	<0.001		-0.32***	<0.001		0.39***	<0.001	
Step 3:			0.01			0.00			0.01
Workload T1 × Workplace resources T1	0.08*	0.032		0.02	0.627		0.04	0.363	
Control T1 × Workplace resources T1	-0.01	0.835		-0.02	0.725		-0.05	0.284	
Reward T1 × Workplace resources T1	0.05	0.216		-0.01	0.869		0.00	0.952	
Community T1 × Workplace resources T1	0.01	0.895		-0.00	0.961		-0.03	0.582	
Fairness T1 × Workplace resources T1	-0.00	0.951		-0.01	0.773		-0.01	0.817	
Values T1 × Workplace resources T1	0.02	0.707		0.06	0.210		0.02	0.663	


*The (standardized) beta values (β) are the coefficients from the finale stage of the analysis; ^∗^*p* < 0.05, ^∗∗^*p* < 0.01, ^∗∗∗^*p* < 0.001.*

**FIGURE 1:**
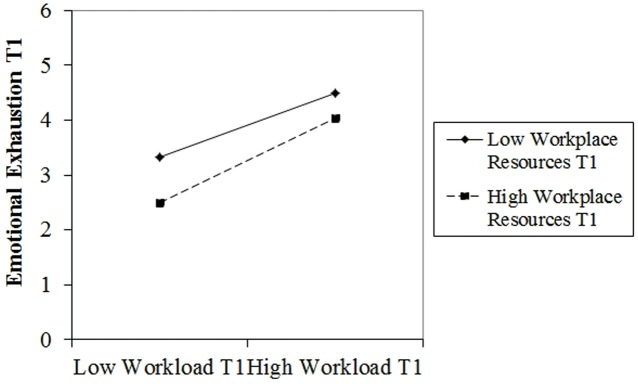
**Two-way interaction between workload (T1) and workplace resources (T1) on emotional exhaustion (T1)**.

Out of the areas of worklife, the areas of reward and values showed to be the most important predictors for all three dimensions of burnout. The area of workload was significant for the criteria emotional exhaustion and cynicism. Workplace resources were negatively related to emotional exhaustion and cynicism and positively related to personal accomplishment. As for the interactions between the areas of worklife and workplace resources, only the interaction between workload and workplace resources was significant for the outcome variable emotional exhaustion. More specifically, the buffering effect of resources seems to work better in an environment with low workload, as low workload combined with high resources has the lowest level of emotional exhaustion (**Figure 1**).

The standardized regression coefficients (β) as well as *p*-values and Δ*R*^2^ are presented in **Table 3**.

### Longitudinal Sample

**Table 4** present the four steps of the analyses. The dependent variables at T1 were stepped into the equation first. This step was significant for all models (Δ*R*^2^_emotional exhaustion_ = 0.54; Δ*R*^2^_cynicism_ = 0.43, Δ*R*^2^_personal accomplishment_ = 0.44). This step was followed by the six areas of worklife (step 2). The first step accounted for an additional variance for cynicism (Δ*R*^2^_cynicism_ = 0.06). The third step included workplace resources, which was non-significant for all outcomes. In the fourth and last step, the interaction terms were included. This step was not significant for all outcomes.

**Table 4:** Hierarchical multiple regression analysis for all predicted variables (T1–T2).

	Emotional exhaustion Time 2			Cynicism Time 2			Personal accomplishment Time 2		
			
Step and variable	β	*p*	ΔR^2^	β	*p*	ΔR^2^	β	*p*	ΔR^2^
Step 1			0.54***			0.43***			0.44***
Dependent variable T1	0.52***	<0.001		0.37***	0.001		0.65***	<0.001	
Step 2: Areas of worklife			0.03			0.06*			0.01
Workload T1	0.17*	0.038		0.03	0.730		0.04	0.646	
Control T1	-0.02	0.828		0.04	0.628		-0.09	0.264	
Reward T1	-0.08	0.356		-0.19*	0.041		0.08	0.380	
Community T1	0.02	0.755		0.11	0.163		-0.05	0.566	
Fairness T1	-0.06	0.505		-0.08	0.421		-0.04	0.712	
Values T1	0.02	0.821		-0.08	0.445		0.03	0.779	
Step 3:			0.00			0.01			0.00
Workplace resources T1	-0.02	0.825		-0.16	0.125		0.15	0.196	
Step 4:			0.02			0.04			0.05
Workload T1 × Workplace resources T1	0.08	0.334		0.00	0.983		0.22**	0.010	
Control T1 × Workplace resources T1	-0.02	0.849		0.05	0.541		0.04	0.686	
Reward T1 × Workplace resources T1	0.09	0.344		0.17	0.094		-0.02	0.840	
Community T1 × Workplace resources T1	0.14	0.087		0.11	0.220		-0.12	0.162	
Fairness T1 × Workplace resources T1	-0.18	0.126		-0.15	0.225		0.12	0.344	
Values T1 × Workplace resources T1	-0.02	0.877		-0.16	0.192		0.15	0.251	


*The (standardized) beta values (β) are the coefficients from the finale stage of the analysis; ^∗^*p* < 0.05, ^∗∗^*p* < 0.01, ^∗∗∗^*p* < 0.001.*

Workload showed to be the most important predictor for emotional exhaustion at T2 and reward was an important predictor for cynicism at T2 (hypotheses 1 and 2). The other areas of worklife did not show significant results with all three dimensions of burnout at T2. Contrary to hypothesis 3, resources at the workplace did not show any direct relationships with all three outcome variables.

**FIGURE 2:**
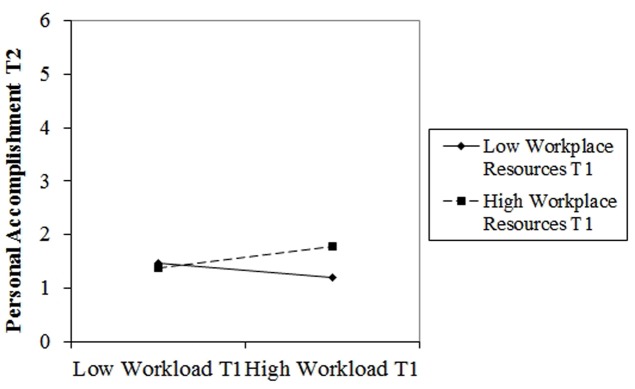
**Two-way interaction between workload (T1) and workplace resources (T1) on personal accomplishment (T2)**.

Out of all interactions, only one was significant: the interaction between workload and workplace resources was significant for personal accomplishment. More specifically, high workload paired with high workplace resources at T1 lead to higher personal accomplishment at T2 (**Figure 2**). As for emotional exhaustion and cynicism, none of the proposed interactions showed significant results.

The standardized regression coefficients (β) as well as *p*-values and Δ*R*^2^ are presented in **Table 4**.

## Discussion

This study explored the relationship between stressors, workplace resources and burnout as the long-term outcome of stress. We investigated which stressors are linked to burnout and if resources are able to buffer the negative effect of stress facets on burnout. The six areas of worklife (Maslach and Leiter, 2008) were used to operationalize stressors. Our hypotheses could be partially verified.

As stated in hypothesis 1, workload is a significant predictor for emotional exhaustion. The relationship between workload on emotional exhaustion is also evident after 6 months, which supports the assumption that high workload causes emotional exhaustion in the long run. High workload has been repeatedly associated with emotional exhaustion (Maslach et al., 2001; Leiter and Maslach, 2009). Constant high workload creates a work environment where employees don’t have time to recover from work demands. This depletes the employees’ capacity to meet work demands and creates long-lasting fatigue and exhaustion. Interestingly, high workload seems to be negatively related to cynicism, indicating that working in an environment with high workload, cynicism is low. However, this effect is no longer visible after 6 months.

The area of reward is related to all dimensions of burnout, but seems to be a strong preventive factor for cynicism even after 6 months. This supports our second hypothesis as well as the findings of Maslach and Leiter (2008). A lack of reward contributes to feelings of inefficacy and meaninglessness which can be precursors for cynicism.

In past research, values showed to be one of the strongest predictors to prevent burnout as it directly affects emotional exhaustion, cynicism, and personal accomplishment (Leiter and Maslach, 2009). In the present study, we could replicate this assumption only in the cross-sectional analysis. Longitudinally, values are not related to burnout after 6 months, therefore hypothesis 3 was only supported in the cross-sectional sample.

Regarding workplace resources, we expected workplace resources to be an important predictor for all three burnout dimensions (hypothesis 4), which was supported in the cross-sectional analyses. However, the direct, positive effect of workplace resources on burnout is not found after 6 months. Furthermore, we expected workplace resources to be a buffer between stressors and burnout (hypotheses 5 and 6). We found a moderating effect of resources for the relationship between workload and emotional exhaustion. In a work environment with low workload, employees can access their resources more easily and thus the buffering effect of resources can take place. However, this effect was very small and was not replicated in the longitudinal sample. The JD-R model (Bakker and Demerouti, 2007), and the resources/recovery-stress model (Kallus, 2016) state that the negative relationship between stress and strain should be weaker if workplace resources are high. However, this buffering effect can only take place if workplace resources are strengthened parallel with increasing stress. In other words, very high stress demands a high recovery of resources to keep the balance (Kallus, 2016). In a work environment with long-lasting stress, workplace resources might be difficult to utilize or might even be depleted to an extent that they cannot be replenished anymore (see also Freedy and Hobfoll, 1994).

In line with hypothesis 6, we found a moderating effect of resources for workload on personal accomplishment. More specifically, high workload seems to be able to enhance personal accomplishment if workplace resources are high. This is in line with the JD-R model, where working in a high workload environment does not have harmful effects when workplace resources are high. In our study, workplace resources even show a beneficial effect, as working in a high workload environment contributes to personal accomplishment when workplace resources are high. The demand-control model (Karasek, 1979) categorizes high demands paired with high resources as “active jobs” and these are characterized with high engagement.

The effects found in the present study are small and do not fully support the buffering effect of workplace resources between stressors and burnout. However, the findings should not be neglected. In the present sample, the participants had much higher values in workload and burnout and lower values in workplace resources compared to the Austrian norm sample. For workload and burnout, we have a “ceiling effect” where significant differences are difficult to detect. Therefore, we suggest interpreting the results in the direction of a buffering effect of workplace resources, although we must point out that this result has to be interpreted with high caution.

In the study of Maslach and Leiter (2008), employees experiencing problems with fairness at the workplace were especially vulnerable to develop burnout over time. Interestingly, our analyses revealed a negative relation between fairness and personal accomplishment at T1, indicating that high fairness at the workplace is associated with lower personal accomplishment. In the simple bivariate correlation, the relationship is positive, though. As we used multiple regression analysis, confounding effects of predictor variables are considered and gives a better representation of the relationship between predictor and outcome. The effect is very small and no longer apparent after 6 months, though. Nevertheless, future studies should be conducted to shed light on this unexpected result.

The area of community was not related to burnout at T2, although research provides evidence that social support at work is related to a lower burnout-risk (e.g., Halbesleben, 2006). The same applies for control. Aspects of control (e.g., autonomy, participation possibilities, latitude) were not directly related to burnout in our study. In their mediation model of job burnout, Leiter and Maslach (2005, 2009) showed that community and control are not directly related to burnout but show indirect relations through values (for community) and through workload (for control). Therefore, all areas of worklife play important roles for burnout, either directly or indirectly through mediation.

### Methodological Issues

An important point of this study lies in the investigation of different types of stress (stressors) instead of using one global indicator of stress. By analyzing different stressors together with different types of resources, the complex interaction between stressors and resources could be investigated much more deeply. In the present study, we used a global indicator of resources as the questionnaire used in this study (RESTQ-Work-27; Jiménez et al., 2016a) only allows building a global score of resources. In future studies, different types of stressors and resources should be assessed to get a deeper understanding in the complex interactions at the workplace.

Same-source bias is a possible limitation of the study. We collected data at different points in time, a practice suggested by Podsakoff and Organ (1986) to reduce the effects of common method bias. Nevertheless, it is possible to take objective indicators for work environment for the analyses (e.g., sickness absences, accident statistics), but these are usually difficult to obtain as companies are hesitant to hand over sensitive data.

As the online survey was declared as “stress study” and open for every interested person, a self-selection bias could occur. Comparing the values of the study sample with a representative sample of Austrian workers, the study sample consists of persons with more burnout and higher stress. This “ceiling effect” has not just drawbacks but also “advantages” in the more conservative sense of research: It is even more difficult to detect significant effects if a ceiling effect is present.

A reason for not finding more longitudinal effects may lie in the time interval of 6 months. It seems plausible to assume that for a health indicator such as burnout, which is considered as relatively stable, permanent changes are not easily achieved but require an exposure over a longer time interval. This assumption is supported by other analyses where effects over a period of 1 year were found (Maslach and Leiter, 2008). Non-significant results also could be due to lack of power because of our small sample size. We therefore emphasize that some coefficients are not significant yet substantial in absolute value, so we do not claim that there is no effect in this case.

### Practical Implications and Conclusion

The findings in the presented study show small effects for the buffering effect of workplace resources on the relationship between stress and the long-term consequence, burnout. In the cross-sectional sample, workplace resources seem to be important prevention factors for burnout, but moderating effects with stressors are still weak. As for the longitudinal effects, more data are needed to investigate this relationship further. Our study includes effects after half a year, but extending the research for longer time periods is needed.

Nevertheless, we stress that workplace resources are important and so it has to be looked on short- and long term effects of resources. In the practical field of risk assessment, assessing workplace resources together with stressors is sometimes overlooked but important to develop sustainable interventions. The stress-strain chain which is the base of risk analysis and the design of workplaces (ISO, 2000b) has to include the stressors as well as buffering aspects especially (Portuné, 2012; Zoni and Lucchini, 2012). For the risk of burnout – which is seen as a long-term impairing effect of stressors (Demerouti et al., 2002; ISO, 2015) – especially the time aspect must be considered in combination with different stressors. Looking at workplaces with high workload for sure the rule of work design means that at first the work environment has to be changed (Portuné, 2012). Especially for these workplaces possible workplace resources should be taken into account when workplace design is considered.

## Ethics Statement

This study was carried out in accordance with the recommendations of the guidelines of the Ethics commission of the University of Graz with written informed consent from all subjects. All subjects gave written informed consent in accordance with the Declaration of Helsinki. The protocol was approved by the Ethics commission of the University of Graz from 02.03.2012.

## Author Contributions

PJ and AD designed the study; conducted research and analyzed the data; AD and PJ wrote and edited the article.

## Conflict of Interest Statement

The authors declare that the research was conducted in the absence of any commercial or financial relationships that could be construed as a potential conflict of interest.
